# Field-scale rice yield estimation based on UAV-based MiniSAR data with Ku band and modified water-cloud model of panicle layer at panicle stage

**DOI:** 10.3389/fpls.2022.1001779

**Published:** 2022-10-06

**Authors:** Zhiyong Wang, Shuli Wang, Hongxiang Wang, Long Liu, Zhenjin Li, Yuandong Zhu, Kai Wang

**Affiliations:** ^1^ College of Geodesy and Geomatics, Shandong University of Science and Technology, Qingdao, China; ^2^ Institute of Intelligent Science and Technology, Shandong Cultural Industry Vocational College, Qingdao, China; ^3^ College of Information Science and Engineering, Shandong Agricultural University, Tai’an, China; ^4^ Laboratory of Target Microwave Properties (LAMP), Zhongke Deqing Academy of Satellite Application (DASA), Huzhou, China

**Keywords:** Rice yield estimation, Modified water-cloud model of panicle layer, Field scale, MiniSAR, Ku band

## Abstract

Scientific and accurate estimation of rice yield is of great significance to food security protection and agricultural economic development. Due to the weak penetration of high frequency microwave band, most of the backscattering comes from the rice canopy, and the backscattering coefficient is highly correlated with panicle weight, which provides a basis for inversion of wet biomass of rice ear. To solve the problem of rice yield estimation at the field scale, based on the traditional water cloud model, a modified water-cloud model based on panicle layer and the radar data with Ku band was constructed to estimate rice yield at panicle stage. The wet weight of rice ear scattering model and grain number per rice ear scattering model were constructed at field scale for rice yield estimation. In this paper, the functional area of grain production in Xiashe Village, Xin'an Town, Deqing County, Zhejiang Province, China was taken as the study area. For the first time, the MiniSAR radar system carried by DJI M600 UAV was used in September 2019 to obtain the SAR data with Ku band under polarization HH of the study area as the data source. Then the rice yield was estimated by using the newly constructed modified water-cloud model based on panicle layer. The field investigation was carried out simultaneously for verification. The study results show: the accuracies of the inversion results of wet weight of rice ear scattering model and grain number per rice ear scattering model in parcel B were 95.03% and 94.15%; and the accuracies of wet weight of rice ear scattering model and grain number per rice ear scattering model in parcel C+D+E were over 91.8%. In addition, different growth stages had effects on yield estimation accuracy. For rice at fully mature, the yield estimation accuracies of wet weight of ear and grain number per ear were basically similar, both exceeding 94%. For rice at grouting stage, the yield estimation accuracy of wet weight of ear was 92.7%, better than that of grain number per ear. It was proved that it can effectively estimate rice yield using the modified water-cloud model based on panicle layer constructed in this paper at panicle stage at field scale.

## 1 Introduction

As one of the three major food corps in the world, rice is an important survival necessity for human beings (Huang et al., 2020). China, as a major rice producer and exporter, ranks first in the world in annual rice output. Scientific and accurate estimation of rice yield is of great significance to national food security and agricultural economic development (Shen et al., 2009; Guo et al., 2020; Huang et al., 2020).

In the face of several unfavorable conditions, such as abnormal global climate change, frequent occurence of natural disaster and continuous population growth, it is an urgent problem to obtain timely and accurate information on rice growth and yield in China.

In the background of continuous development of science and technology, intelligent methods for monitoring rice gradually appear (Gu et al., 2022; Huang et al., 2022). With the development of satellite remote sensing technology and the improvement of agricultural remote sensing level, it has become a scientific and technological method of modern agriculture to monitor rice growth and estimate rice yield using remote sensing technology. At present, optical remote sensing, hyperspectral remote sensing, microwave remote sensing (including microwave scatterometer, synthetic aperture radar) and other remote sensing techniques have been successfully applied to monitor the rice growth and yield estimation (Jia et al., 2014; Guan. K et al., 2018; Setiyono et al., 2018; Wu et al., 2020; Alebele et al., 2021; Jing et al., 2022).

Different types of sensors also have their own advantages and disadvantages in rice yield estimation. Because rice is mainly grown in the cloudy and rainy tropical and subtropical regions, it is often covered by cloud and rain for a long time during its growth cycle. Such as Zhejiang province, Hunan province, Hubei province, Guangdong province and other provinces in South China, during the growth cycle of early rice, the probability of obtaining an optical remote sensing image with cloud amount less than 10% is only 7%. As a result, it is often influenced by weather when monitoring the rice by optical satellite remote sensing (Shen et al., 2009; Jia et al., 2014; Huang et al., 2020). This has limited the large-scale application and promotion of related yield estimation methods. Synthetic Aperture Radar (SAR) is not influenced by cloud, fog, rain, snow and other weather, and it can obtain image data with the advantages of day/night data acquisition, all-weather imaging capability, and strong penetrability (Li et al., 2022; Wang et al., 2022; Yu et al., 2022). Satellite remote sensing can observe the earth from space over a large area. At present, radar remote sensing has become one of the best observation techniques for monitoring the rice and yield estimation (Shen et al., 2009; Jia et al., 2014; Huang et al., 2020; Wu et al., 2020). At the same time, radar remote sensing technology can obtain the radar response characteristics of rice canopy under different polarization, including scattering intensity information and phase information, which can better reflect the rice canopy water content, plant structure and growth situation (Guan. K et al., 2018; Setiyono et al., 2018; Guo et al., 2020). Radar remote sensing is complementary to optical remote sensing. So it can provide abundant data support for establishing reliable and stable rice monitoring system based on radar remote sensing.

The methods and applications for monitoring rice and yield estimation based on radar remote sensing technology have been studied by many researchers. At present, radar remote sensing technology has been successfully applied in monitoring the rice planting area and mapping the rice classification (Yang et al., 2017; Yuzugullu et al., 2017; Guan. K et al., 2018; Guo et al., 2018; Mandal et al., 2018; Alebele et al., 2020; Dipankar et al., 2020; Yang et al., 2021). On this basis, many researchers began to focus on rice plant height inversion (Lee et al., 2018; Ndikumana et al., 2018; Guo et al., 2020) and rice yield estimation (Shen et al., 2009; Zhang et al., 2017; Kersten et al., 2018; Asilo et al., 2019; Huang et al., 2020), and achieved a series of achievements. Traditional SAR rice monitoring and yield estimation are often based on the SAR data with low frequency, such as X, C band and L band. Compared with the SAR data with high frequency, it has certain difficulties when usig SAR data with low frequency to estimate the rice yield: microwave with low frequency band could penetrate rice panicle layer, which makes the radar echo containing much information about stem and leaf layer and even the underlying surface. This increases the difficulty of the modeling, and reduces the yield estimation accuracy. To solve this problem, Jia et al. (2014) firstly established a forward microwave scattering model of rice, which contained a large number of parameter information on stem and leaf layer and the underlying surface, and then constructed inversion model based on neural network to establish rice yield model. However, if more radar echoes of rice canopy are derived from rice panicle layer, the number of input parameters in forwarding modeling can be greatly reduced.

To overcome the disadvantages of radar data with low frequency when estimating the rice yield, the SAR data with high frequency can be used for rice yield estimation. At present, researches on monitoring the rice by SAR data with high frequency have been carried out internationally. As early as 1989, Toan et al. (1989) used airborne SAR in France to obtain dual-polarization and multi-temporal radar images of rice in X band during the growing stage and extracted radar backscattering characteristics. The study found that before rice was at tillering stage, the backscattering coefficient increased with the growth of rice, showing a strong correlation with the biomass of rice. This is the first application of SAR data with high frequency in monitoring rice. In 2000, Kim et al. (2000) used X band scatterometer to study the change of backscattering coefficient over time in rice fields, and obtained the continuous response value of incident angle (0-70°) for the first time. The study showed that the backscattering coefficient reached the maximum of about 43-60 days after rice transplanting. In 2002, Inoue et al. (2002) analyzed the relationship between backscattering coefficients of different frequencies and rice growth parameters by using multi frequency and full polarized scatterometer, and found that the backscattering coefficients of high frequency microwave (Ka, Ku, X band) were highly correlated with weight of ear. It provided a basis for ground measurement of rice panicle biomass inversion based on SAR data with high frequency. From 2013 to 2014, further experimental studies (Inoue and Sakaiya, 2013; Inoue et al., 2014) found that the backscattering coefficient under VV polarization decreased with the increase of panicle grain plumpness at the filling stage. The sensitivity of backscattering coefficient under VV polarization to the biomass of rice ear was explained experimentally, and the radar data with X band was significantly correlated with weight of ear at high incidence angle. In 2012, Nguyen et al. (2012) selected TerraSAR-X data of five time phases after rice sowing to perform linear regression fitting on rice field measurement data to estimate rice yield. Compared with official statistical data, the fitting accuracy of total output is up to 95%. The results showed that the high resolution X band SAR data is reliable in rice yield estimation. From 2015 to 2017, based on the high frequency dual polarization TanDEM-X data, Lopez-Sanchez, Erten, and Rossi et al. (Erten et al., 2015; Rossi and Erten, 2015; Erten et al., 2016; Lopez-Sanchez et al., 2017) monitored the changes of rice plant height during the growing stage, verified its potential in rice plant height inversion, and provided effective data support for rice yield estimation. Maki et al. (2017) used Cosmo-SkyMed data and rice leaf area index integrated crop model (SIMRIW-RS) to estimate regional rice yield in 2017. In conclusion, it is an effective data source to estimate rice yield using SAR data with high frequency. However, since the spaceborne SAR systems mostly work in X band or C band, it is still to be studied the feasibility and advantages of Ku band and other high frequency bands in rice yield estimation.

The water-cloud model is an effective model proposed by Attema and Ulaby in 1978 (Attema and Ulaby, 1978). On this basis, many modified water-cloud models have been proposed (Liu et al., 2012; Yang et al., 2016; Setiyono et al., 2019; Wu et al., 2020), such as the two layer water-cloud model. At present, rice yield estimation based on water-cloud model has achieved good results. Yang et al. (2016) estimated the change of wheat by using the modified water-cloud model (MWCM) and multi-temporal RADARSAT-2 images. The validation results showed that the MWCM could predict the temporal behaviors of the rice variables well during the growth cycle (R^2^ >0.8). Setiyono et al. (2019) used SAR images and ORYZA crop growth model to realize the estimation of rice yield in a large area in South and Southeast Asian countries. Based on a single TerraSAR image, Wu et al. (2020) explored the effects of water-cloud model with different layers on rice yield estimation, indicating that single-layer water-cloud model is better than a double-layer water-cloud model in grain number estimation. In later developments, rice yield estimation based on remote sensing images also began to be combined to computer science, including physical scattering model, optimization algorithm, and gradient regression (Zhang et al., 2020; Arumugam et al., 2021). The results are better than those of the original models.

It can provide data support for fine agriculture research to realize the rice yield estimation at the field scale (Wang et al., 2019). At present, the Unmanned Aerial Vehicle (UAV) remote sensing has been widely used in rice yield estimation research work in field scale or small scale, but most of them are used the optical sensors or hyperspectral sensors (Zheng et al., 2018; Duan et al., 2019; Wang et al., 2019; Wang et al., 2022). Now, the research for rice yield estimation using UAV-based SAR data is very few, especially for the high frequency band, such as Ku band. When radar operates in Ku band, the backscattering is mainly from panicle layer. So the radar with Ku band is an effective sensor for rice yield estimation. It has advantages of convenience and maneuverability to estimate the rice yield using UAV-based MiniSAR. The research on rice yield estimation using UAV-based MiniSAR can enrich the technical approach to rice yield estimation at the field scale and it can provide a new means of rice yield estimation.

To verify the potential of radar data with Ku band in rice yield estimation and realize rice yield estimation at field scale, based on the radar data with Ku band acquired by the MiniSAR radar system mounted on the UAV platform, this paper constructed a rice yield estimation model suitable for the radar data with Ku band at panicle stage, and carried out rice yield estimation based on the backscattering coefficient of high-frequency SAR data and a modified water-cloud model.

The structure of this paper is arranged as follows: It introduces the study area and data in section 2. It mainly introduces the acquisition of UAV-based MiniSAR data, which can provide the radar data with Ku band. Section 3 is the method of this paper. In order to realize rice yield estimation at the field scale, a new modified water-cloud model based on panicle layer is constructed for rice yield estimation at panicle stage. It includes the wet weight of rice ear scattering model and grain number per rice ear scattering model. Section 4 is the experiment and results of this paper. The experiment is introduced in detail. Section 5 is the discussion and analysis part. Finally, some important conclusions of this study are given.

## 2 Study area and materials

### 2.1 Study area

**Figure 1:**
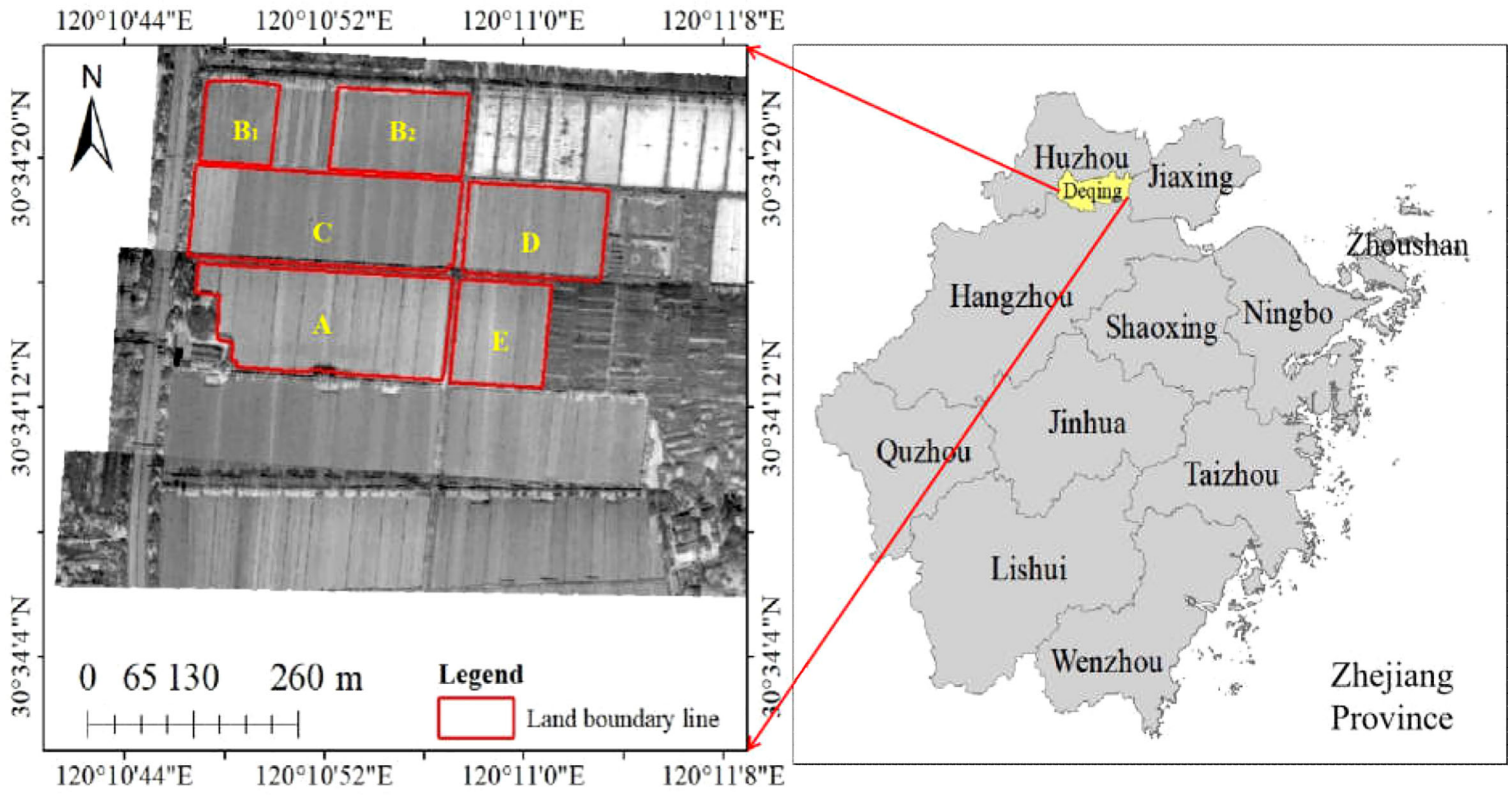
Geographical map of the study area. The left image shows the MiniSAR radar images and the locations of **(A–E)** parcels in the study area. The maps on the right show the geographical location of the study area.

The study area is located in the the functional area of grain production in Xiashe Village, Xin 'an Town, Deqing County, Zhejiang Province, China, which covers an area of 1 km^2^. Its longitude ranges from 120°C10′40′′E to 120°C11′15′′E, and its latitude ranges from 30°C34′00′′N to 30°C34′25′′N, as shown in **Figure 1**. The study area is located in the HangJiaHu plain with the fertile land and belongs to grain mulberry area. It is also known as "land of fish and rice" and "home of silk". The study area has a subtropical humid monsoon climate, warm and humid, with distinct four seasons. The annual average temperature is 13-16°C, and the annual average precipitation is about 1379 mm, which are suitable for single-season rice growth. The functional area is dominated by rice and rape, with rice growing season from early June to the middle of November and rape growing season from November to the following May.

The main types of rice planted in the study area are Nanjing 46 and Nanjing 9108. Nanjing 46 is about 110 cm in plant height, compact plant type, medium with strong in tillering, large panicle type, upright panicle length of about 15cm, total grain number of 140-150 per panicle, setting percentage more than 90%, and 25-26 g per 1000 grains. Nanjing 9108 is about 96.4 cm in plant height, compact plant type and strong in tillering, total grain number of 125.5 per panicle, setting percentage about 94.2%, and 26.4 g per 1000 grains.

### 2.2 Data

#### 2.2.1 MiniSAR data

MiniSAR is a radar system for UAVs independently developed by the Academy of Aerospace Information Innovation, Chinese Academy of Sciences (CAS). The radar system operates in the Ku band with the frequency of 14.6GHz and HH polarization. The undercenter look angle and incidence angle of the UAV-based MiniSAR image used in this study are both 50°. The MiniSAR radar system carried by the DJI M600 UAV platform was used to photograph the rice in the study area, obtain high resolution SAR images, and complete the acquisition of radar data of rice field. For the UAV-based MiniSAR data, we carried out speckle noise filtering, the radar backscatter information was presented in dB scale. The spatial resolution of the UAV-based MiniSAR image used in the experiment is 0.3m*0.3m. The area of the randomly selected sampling plot in the rice field survey sampling is 1.5m×1.5m, which theoretically includes 5*5 pixels in the SAR image. The backward scattering coefficients of 5*5 pixels are averaged to one when estimating the rice yield.

On the day of the experiment, the weather was clear with the north wind of level 1-2. The UAV was manually controlled to take off, and the flight altitude was 150 meters. The first flight was from 14:00 to 16:00 on September 24, 2019, and the second and third flights were from 9:00 to 11:00 on September 25, 2019, respectively. The acquisition of UAV-based MiniSAR data is about a week after the rainfall. Therefore, the research results of this paper are also basically not affected by seasonality. The flight range covered the whole rice study area. **Table 1** shows the working parameters of the MiniSAR system. In the study area, five parcels (named parcel A, B, C, D and E) were selected for research and analysis.

**Table 1:** The parameters of UAV-based MiniSAR data.

Item	Product Level
**UAV platform**	DJI M600
**Sampling mode**	Intermediate frequency sampling
**Operating mode**	Single channel
**Radar band**	Ku
**Polarization**	HH
**Resolution (m)**	0.3× 0.3
**Center frequency (GHz)**	14.6
**Pulse bandwidth (MHz)**	1200
**Side direction**	Right
**Undercenter look angle (°)**	50
**Beam angle in range direction (°)**	40
**Beam angle in azimuth direction (°)**	6

#### 2.2.2 Field investigation data

**Figure 2:**
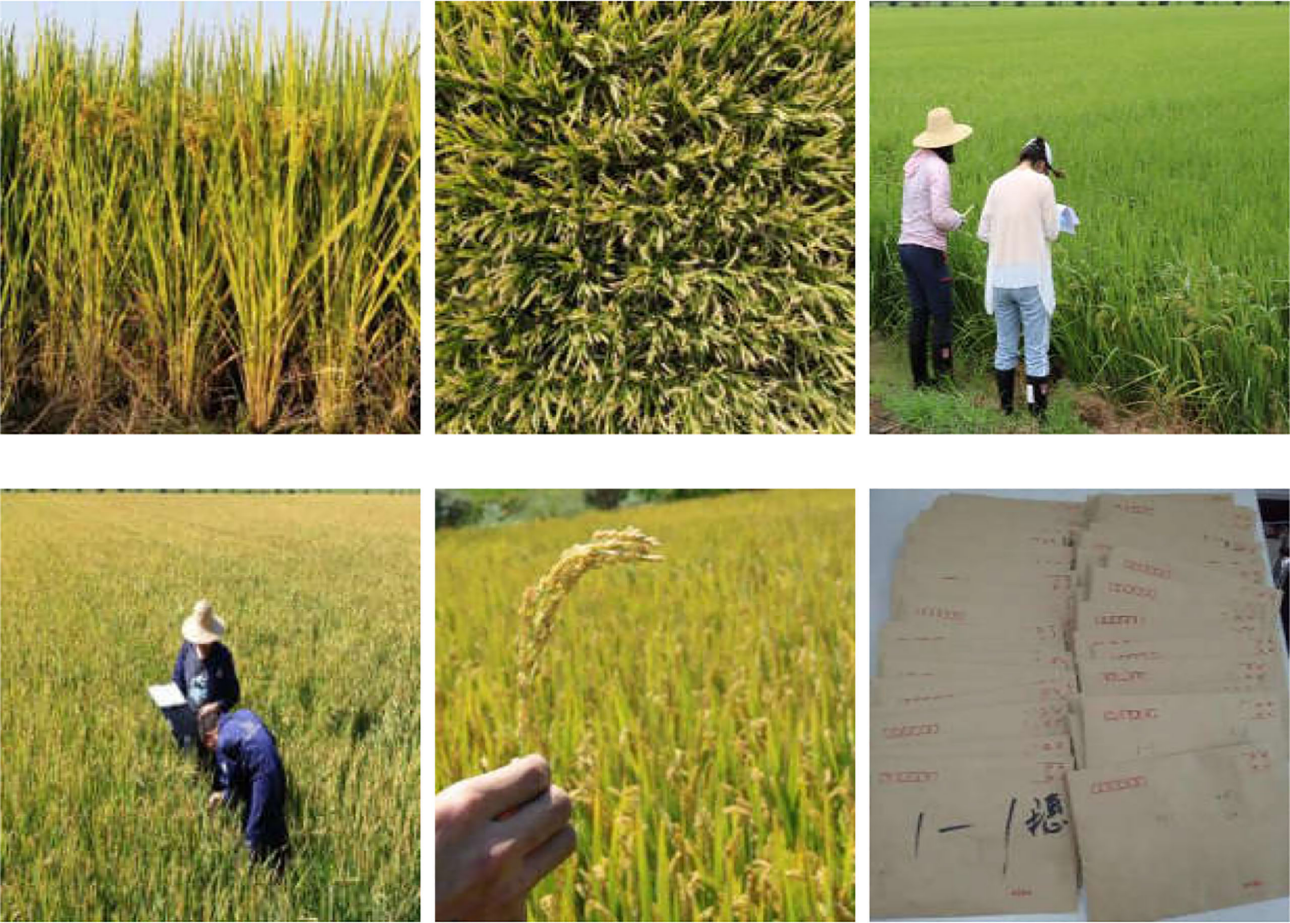
Photos taken during the field investigation in the study area.

Field samples were collected from parcels B, C, D and E in the study area from September 23 to 25, 2019. Parcel B and parcel C are large, with an area of 22,759.4m^2^(about 2.28 hectares) for parcel B and 24,579.35m^2^(about 2.46 hectares) for parcel C. 40 sampling plots with an area of 1.5m×1.5m were randomly selected in parcel B and parcel C, and 40 rice pancel samples were collected, and numbered from 1 to 40. As a small area in the middle of parcel B is used for nitrogen fertilizer experiment, the amount of fertilizer application is different from other plots, and two varieties of Nanjing 46 and Nanjing 9108 are planted respectively, so this part of sampling is not carried out. Instead, rice fields outside this small area are selected, namely areas parcel B_1_ and parcel B_2_. All rice varieties in this area are Nanjing 9108 with uniform growth. Data collected in the two parcels were used for modeling and later validation. For parcel B, 40 original samples were collected, 28 samples were available after data cleaning, 18 were randomly selected for modeling, and the remaining 10 samples were used as validation data. For parcel C, 40 original samples were collected. The area of parcel D and parcel E is small, parcel D covers an area of about 12,763.98m^2^(about 1.28 hectares), and parcel E covers an area of about 9,493.61m^2^(about 0.95 hectares). 25 sampling plots with an area of 1.5m×1.5m were randomly selected in parcel D, 25 sampling plots with an area of 1.5m×1.5m were randomly selected in parcel E. 50 rice panicle samples were collected in parcel D and parcel E, and numbered from 1 to 50. Considering that the sowing and heading dates of rice in parcels C, D, and E were similar, the samples of the three parcels were combined to the parcel C+D+E to conduct yield estimation modeling and yield estimation. So for parcel C+D+E, a total of 90 original samples were collected. 10 samples were available for each parcel C, D and E after data cleaning, so a total of 30 samples for parcel C+D+E, and then 20 samples were randomly selected for modeling and the remaining 10 samples were used for validation. **Figure 2** is some photos taken during the field investigation.

## 3 Method

**Figure 3:**
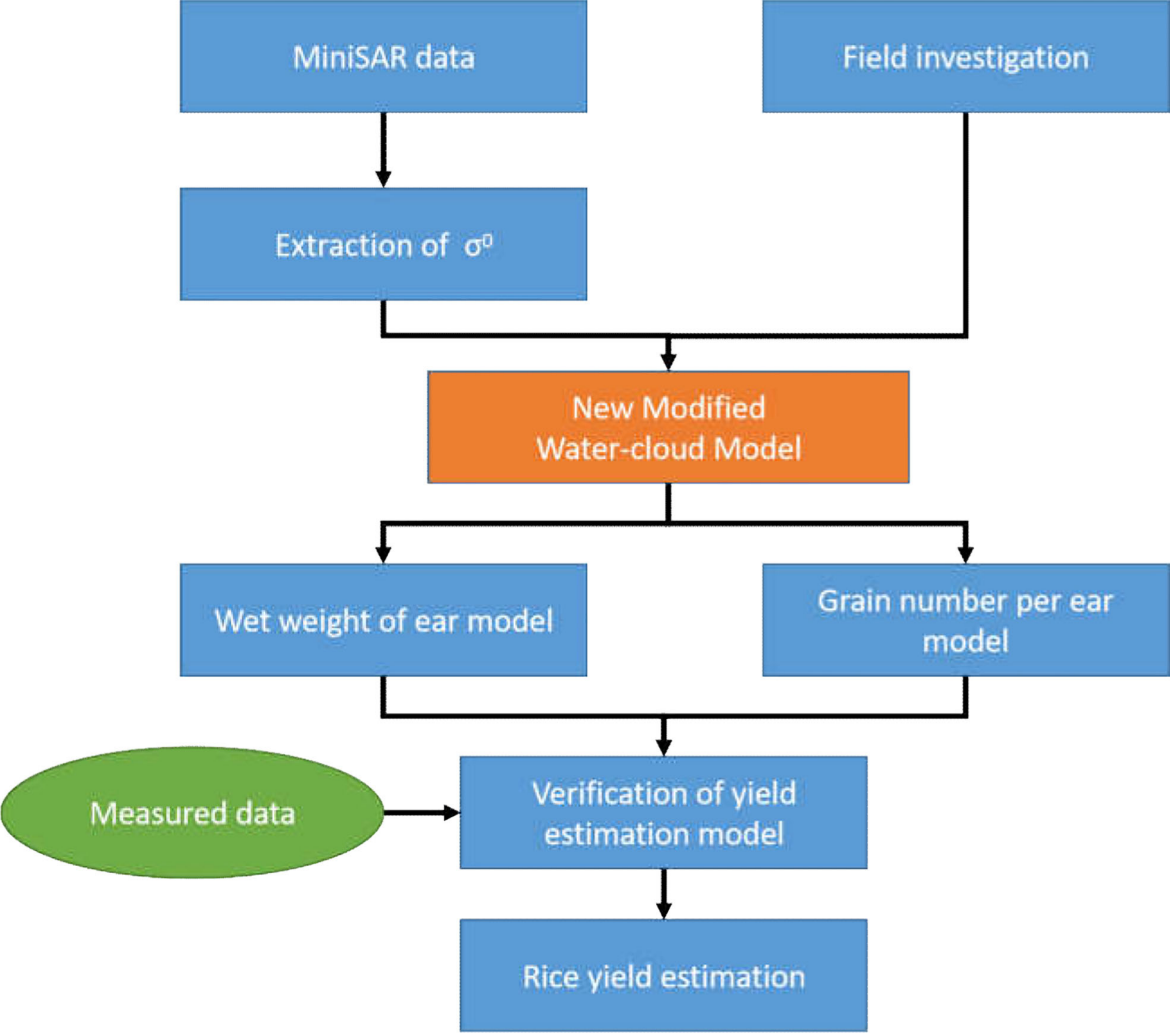
Overall technical flow chart.

To solve the problem of rice yield estimation at the field scale, we constructed a rice yield estimation method based on a new modified water-cloud model at panicle layer and the radar data with Ku band. Using the MiniSAR system mounted by DJI M600 UAV as the sensor, the synthetic aperture radar (SAR) data with Ku band during panicle stage in the study area were obtained, and the parameters of yield estimation model and rice yield in the study area were inverted. The overall technical flow chart of this paper is shown in **Figure 3**.

### 3.1 New modified water-cloud model based on panicle layer

The semi-empirical water-cloud model was firstly proposed by Attema and Ulaby et al. (Attema and Ulaby, 1978) in 1978. The water cloud model is suitable for rice biomass inversion because of its simple structure, fewer parameters and easy to get the reverse solution.

**Figure 4:**
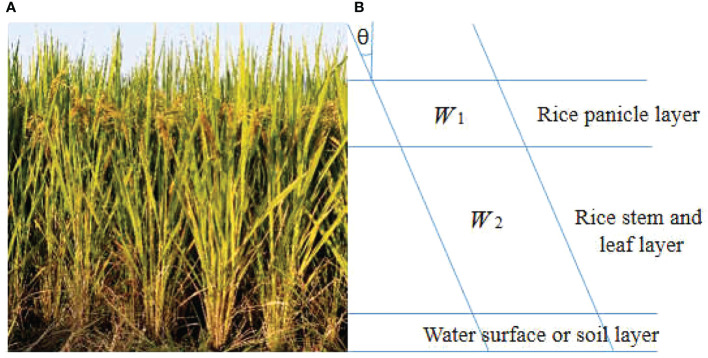
The simulation figure of the newly constructed water-cloud model based on panicle layer. **(A)** is the actual scene of the rice plant. **(B)** is the schematic diagram of new model. The canopy of rice is mainly divided into two parts: the pancicle layer, stem and leaf layer.

Yang et al. (2016) proposed the Modified Water-Cloud Model (MWCM), which considered phenology information and canopy level heterogeneity. Because the backscattering of high frequency band radar such as Ku band is mainly from the panicle layer of rice, n this paper, inspired by the MWCM model, the rice canopy is divided into two layers: panicle layer and stem and leaf layer, in order to obtain more accurate wet biomass of rice ear. **Figure 4** is the simulation figure of the new water-cloud model based on panicle layer.

The expression of unit volume water content W (kg/m^3^) of rice canopy can be expressed as follows:


(1) $$\text{W}={\text{W}}_{1}+{\text{W}}_{2}$$

(2) $$W_{1}=(m_{1w}-m_{1d})\cdot n/h_{1}$$

(3) $$W_{2}=({\text{m}}_{2w}-{\text{m}}_{2d})\cdot \text{n}/{\text{h}}_{2}$$

Where, *W*_1_ and *W*_2_ are water content per unit volume (kg/cm^3^) of panicle layer and stem and leaf layer; *h*_1_ and *h*_2_ are height (m) of panicle layer and stem and leaf layer; *m_1w_* and *m_2w_* are wet weight (kg/plant) of panicle layer and stem and leaf layer for single plant respectively; *m_1d_* and *m_2d_* are dry weight (kg/plant) of panicle layer and stem and leaf layer for single plant respectively. Parmater *n* is the number of plants per unit area (plants/m^2^).

The penetration of microwave with high frequency is poor. It is difficult to reach the ground through the stem and leaf layer. In general, for the total backscattering from the whole vegetation canopy, the proportion of multiple scattering from soil and vegetation canopy is very small. So we do not consider this kind of scattering when constructing new modified water-cloud model. Therefore, the following formula can be used to describe the total backscattering from rice canopy:


(4) $$\sigma^{0}={\sigma^{0}}_{ear}+{\tau^{2}}_{ear}\cdot {\sigma^{0}}_{s\&l}+{\tau^{2}}_{ear}\cdot {\tau^{2}}_{s\&l}\cdot {\sigma^{0}}_{soil}$$

Where, *σ* ^0^ is the total backscattering from rice canopy; *σ* ^0^ *ear* is the volume scattering from rice panicle layer; *σ* ^0^ *s*&*l* is the volume scattering from rice stem and leaf layer; *σ* ^0^ *soil* is the radar scattering reflected by soil after the attenuation of canopy; *τ* ^2^ *ear* and *τ* ^2^ *s*&*l* are the bidirectional attenuation coefficients of panicle layer and stem and leaf layer, and the canopy and soil layer respectively. The calculation formula of *τ* ^2^ *ear* and *τ* ^2^ *s*&*l* are respectively expressed as:


(5) $${\tau^{2}}_{ear}=\exp(-2N_{1}\cdot Q\cdot h_{1}/\cos\theta )$$

(6) $${\tau^{2}}_{s\&l}=\exp(-2N_{2}\cdot Q\cdot h_{2}/\cos\theta )$$

Where, *θ* is the incidence angle of radar beam; N_i_(i = 1 or 2) represents the number of water droplets in panicle layer, stem and leaf layer per unit volume respectively; and Q represents the attenuation cross section of a single water droplet.

Assuming that *α* denotes the attenuation coefficient of radar waves within the canopy and *η* denotes the radar cross section per unit volume in the vegetation canopy, which are defined as:


(7) $$\eta =N_{i}\cdot \lambda$$

(8) $$\alpha =N_{i}\cdot Q$$

Where *λ* is the scattering cross section of a single water droplet particle.

The volume scatterings from panicle layer and stem and leaf layer of rice are respectively expressed as:


(9) $${\sigma^{0}}_{ear}=(\lambda /2Q)[1-\exp(-2N_{1}\cdot Q\cdot h_{1}/\cos\theta )]$$

(10) $${\sigma^{0}}_{s\&l}=(\lambda /2Q)[1-\exp(-2N_{2}\cdot Q\cdot h_{2}/\cos\theta )]$$

The backscattering coefficient from soil is expressed as:


(11) $${\sigma^{0}}_{soil}=A\exp(B\cdot m_{s})$$

Where, parameter A and B represent two parameters related to radar band, incidence angle, polarization mode and ground roughness; parameter *λ* represents the scattering cross section of a single water droplet; parameter *m_s_* represents the water content per unit volume of soil.

Finally, considering the attenuation effect of rice canopy on radar wave, the new modified water-cloud model based on rice panicle layer is constructed in this paper as follows:


(12) $$\begin{array}{l} \sigma^{0}=(\lambda /2Q)[1-\exp(-2N_{1}\cdot Q\cdot h_{1}/\cos\theta )]\\ +(\lambda /2Q)[1-\exp(-2N_{2}\cdot Q\cdot h_{2}/\cos\theta )]\exp(-2N_{1}\cdot Q\cdot h_{1}/\cos\theta )\\ +A\exp(B\cdot m_{s})\exp(-2N_{1}\cdot Q\cdot h_{1}/\cos\theta )\exp(-2N_{2}\cdot Q\cdot h_{2}/\cos\theta ) \end{array}$$

For ease of expression, we replace (7) with the parameter C. Since N is proportional to W, we replace NQ with DW, and the expression becomes:


(13) $$\begin{array}{l} \sigma^{0}=C[1-\exp({-2DW}_{1}\cdot h_{1}/\cos\theta )]\\ +C[1-\exp(-2DW_{2}\cdot h_{2}/\cos\theta )]\exp(-2DW_{1}\cdot h_{1}/\cos\theta )\\ +A\exp(B\cdot m_{s}-2DW_{1}\cdot h_{1}/\cos\theta -2DW_{2}\cdot h_{2}/\cos\theta ) \end{array}$$

The above equation is simplified as:


(14) $$\begin{array}{l} \sigma^{0}=C[1-\exp(-2DW_{1}\cdot h_{1}/\cos\theta -2DW_{2}\cdot h_{2}/\cos\theta )]\\ +A\exp(B\cdot m_{s}-2DW_{1}\cdot h_{1}/\cos\theta -2DW_{2}\cdot h_{2}/\cos\theta ) \end{array}$$

σ^0^ can also be expressed in decibels (dB) as:


(15) $$\begin{array}{l} \sigma^{0}=10\cdot {\text{log}}_{10}\{C[1-\exp(-2DW_{1}\cdot h_{1}/\cos\theta -2DW_{2}\cdot h_{2}/\cos\theta )]\\ +A\exp(B\cdot m_{s}-2DW_{1}\cdot h_{1}/\cos\theta -2DW_{2}\cdot h_{2}/\cos\theta )\} \end{array}$$

In the formula, the model parameters are denoted by A, B, C and D, which are obtained by regression analysis and fitting of the model to the measured rice backscatter coefficients. In some studies, the model coefficients A, B and D also are obtained by simulation with the soil backscatter model.

The study by Yang et al. (Shenbin, 2008) pointed out that there are two forms of rice water cloud models: (1) the rice water cloud model with the water layer as a special soil treatment; (2) the rice water cloud model based on the scattering mechanism. In this paper, we choose the model that is easier to derive its inverse function, the "rice water cloud model with the water layer as a special soil treatment" to carry out the inversion of rice biological parameters and complete rice yield estimation at the panicle stage.

During the maturity of rice, the depth of the water layer in the paddy field is usually 2 cm-5 cm, and some parcels are not covered by the water layer, and the soil of the paddy field is wet. Moreover, the penetration of high-frequency SAR microwaves is small and it is difficult to penetrate the stem and leaf layer to reach the ground, so the improved water cloud model for the rice panicle layer in the paper is the Equation (10).


(16) $$\begin{array}{l} \sigma^{0}=C(1-\exp(-2DW_{1}\cdot h1/\cos\theta -2DW_{2}\cdot h_{2}/\cos\theta ))\\ +{\sigma^{0}}_{BG}\exp(-2DW_{1}\cdot h_{1}/\cos\theta -2DW_{2}\cdot h_{2}/\cos\theta ) \end{array}$$

Formula (16) can be simplified as follows:


(17) $$\sigma^{0}=C+({\sigma^{0}}_{BG}-C)(\exp(-2DW_{1}\cdot h_{1}/\cos\theta -2DW_{2}\cdot h_{2}/\cos\theta ))$$

Where, *σ* ^0^_*BG*_ is a constant term, representing the backscattering coefficient of the water layer covering rice field. For the new modified water-cloud model, *h*_1_ and *h*_2_ represent the heights of rice panicle layer and stem and leaf layer; W_1_ and W_2_ represent the water content per unit volume of rice panicle layer and stem and leaf layer. Therefore, W_1_·h_1_ and W_2_·h_2_ represent the water content per unit area of rice panicle layer and stem and leaf layer respectively.

Formula (17) is the new modified water-cloud model based on panicle layer constructed in this paper for rice yield estimation at panicle stage, which is mainly suitable for the radar data with high frequency, such as Ku band. Considering that the Ku band is less penetrating, it will be more sensitive to the rice panicle layer. It is less sensitive to the stem and leaf layer, which affect the sensitivity of parameters such as leaf area index (LAI) and plant height. Therefore, the improved water cloud model in this paper was simplified by not considering information on other parameters affecting the model, the sensitivity analysis was not performed for the relevant parameters, and only considering the rice panicle layer.

### 3.2 Estimation of the model parameters

There are only three unknown parameters in the formula (17): C, D and *σ* ^0^_*BG*_. The above parameters are usually obtained by fitting method after field measurement of backscattering coefficient and water content of rice.

By combining the similar terms of formula (17), we can obtain the formula (18) as follows:


(18) $$\left(W_{1}\cdot h_{1}+W_{2}\cdot h_{2})=\frac{\cos\theta }{-2D}\ln(\frac{\sigma^{0}-C}{{\sigma^{0}}_{BG}-C}\right)$$

Suppose that the parameter C satisfies C>σ^0^, then $\text{C}>{\text{σ}}_{\text{BG}}^{0}$ . We can get:


(19) $$a=\frac{\cos\theta }{2D}\ln(C-{\sigma^{0}}_{BG})$$

(20) $$b=\frac{\cos\theta }{2D}$$

Then the final inversion formula is:


(21) $$\left(W_{1}\cdot h_{1}+W_{2}\cdot h_{2})=a-b\ln(C-\sigma^{0}\right)$$

Its inverse function is:


(22) $$\sigma^{0}=C-\exp(-((W_{1}\cdot h_{1}+W_{2}\cdot h_{2})-a)/b)$$

Where, the parameter *a* and *b* represent the coefficients of the inversion formula; the parameter *C* represents the volume scattering coefficients when rice sealing line; the parameter *W_1_*, *W_2_*, *h_1_* and *h_2_* are obtained through field measurement experiments.

## 4 Results

### 4.1 Experimental results

Generally, in the relatively mature stage of rice, except for the dominant leaves, the scattering intensity of rice canopy is largely derived from the scattering of rice panicle. For rice field, the more ears of rice, the more grain number per ear. Panicle density (the number of ears of rice per square meter) and panicle length are also directly related to grain yield (Lee et al., 2018).

For parcel B, 18 samples from field investigation were randomly selected for modeling. For parcel C+D+E, 20 samples from field investigation were randomly selected for modeling.

**Figure 5:**
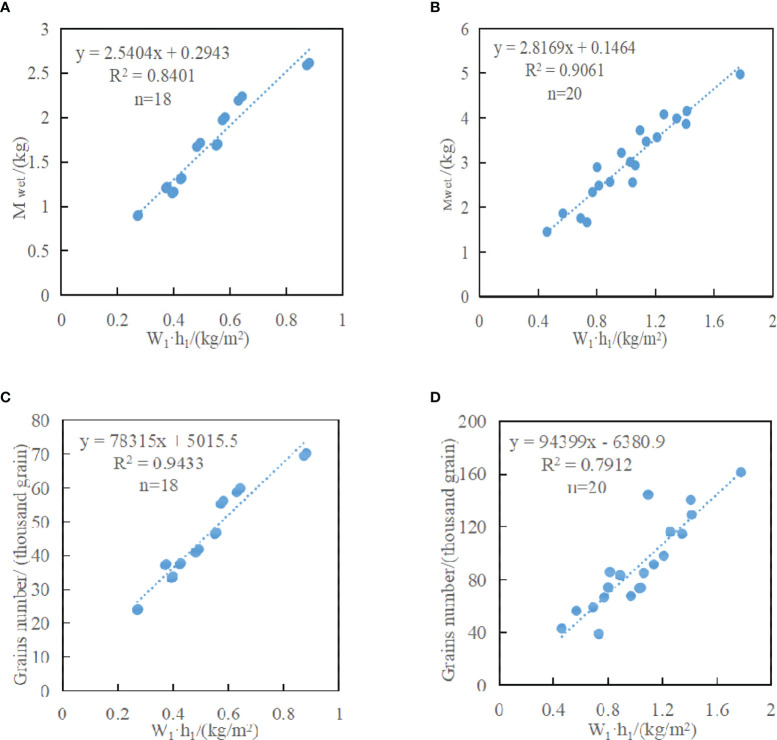
Inversion model of wet weight of ear and grain number per ear. **(A)** is the scatter diagram between (W_1_·h_1_)_B_ and wet weight of rice ear scattering model M_wetB_. **(B)** is the scatter diagram between (W_1_·h_1_)_CDE_ and wet weight of rice ear scattering model M_wetCDE_. **(C)** is the scatter diagram between (W_1_·h_1_)_B_ and grain number per rice ear scattering model N_B_. **(D)** is the scatter diagram between (W_1_·h_1_)_CDE_ and grain number per rice ear scattering model N_CDE_.

Based on the inversion formula of W_1_·h_1_, wet weight of ear and grain number per ear of rice can be inverted. Statistical analysis of relevant experimental data obtained in this paper showed that W_1_·h_1_ had a linear relationship with wet weight of ear and grain number per ear of rice. **Figure 5** is the inversion model of wet weight of ear and grain number per ear of rice, and formula (23)~(26) are the inversion model formulas.

The model of wet weight of ear of rice in parcel B and parcel C+D+E are as follows:


(23) $$M_{wetB}=2.5404\times {\left(W_{1}\cdot h_{1}\right)}_{B}+0.2943\begin{array}{c} {R^{2}}_{B}=0.8401 \end{array}$$

(24) $$M_{wetCDE}=2.8169\times {\left(W_{1}\cdot h_{1}\right)}_{CDE}+0.1464\begin{array}{c} {R^{2}}_{CDE}=0.9061 \end{array}$$

The model of grain number per ear of rice in parcel B and parcel C+D+E are as follows:


(25) $$N_{B}=78315\times {\left(W_{1}\cdot h_{1}\right)}_{B}+5015.5\begin{array}{c} {R^{2}}_{B}=0.9433 \end{array}$$

(26) $$N_{CDE}=94399\times {\left(W_{1}\cdot h_{1}\right)}_{CDE}-6380.9\begin{array}{c} {R^{2}}_{CDE}=0.7912 \end{array}$$

Where, *M*_wetB_ and *M*_wetCDE_ are respectively the wet weight of ear per square meter (kg/m^2^) of rice in parcel B and parcel C+D+E; *N*_B_ and *N*_CDE_ are respectively the grain number per ear per square meter (thousand grains/m^2^) of rice in parcel B and parcel C+D+E; R^2^_B_ and R^2^_CDE_ are the model correlation coefficients respectively.

The results of **Figure 5** showed that there were two high linear correlations of rice between wet weight of ear and water content per unit area (W_1_·h_1_), and between grain number per ear and water content per unit area (W_1_·h_1_). By observing **Figures 5A, B**, it can be found that under HH polarization, the correlation between W_1_·h_1_ and wet weight of ear of rice in parcel B (**Figure 5A**) was much smaller than that in parcel C+D+E (**Figure 5B**). The reason for the difference in correlation was that the rice in parcel B was fully mature and about to be harvested, with less water content in panicle. The rice in parcel C+D+E was in the grouting stage with more water content. Similarly, under HH polarization, the correlation between W_1_·h_1_ and grain number per ear was more than 0.94 in parcel B (**Figure 5C**), but the result of parcel C+D+E (**Figure 5D**) was far worse than that of parcel B (**Figure 5C**). According to the analysis, the difference of rice varieties planted in parcel C+D+E leads to the difference in the grain number per ear, resulting in uneven distribution of panicle grains per unit area. However, the rice varieties in parcel B are all Nanjing 9108, with uniform growth. The above analysis shows that it is feasible and effective to estimate the rice yield using the new modified water-cloud model based on panicle layer constructed in this paper.

**Figure 6:**
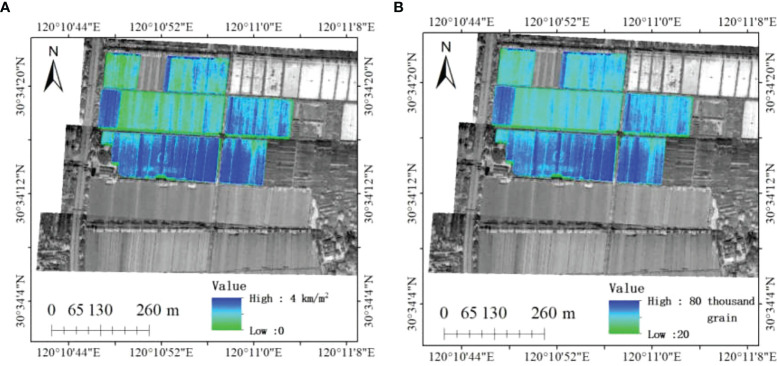
The estimation yield map based on the new modified water-cloud model of panicle layer. **(A)** is the estimation values based on M_wetB_ model of wet weight of rice ear; **(B)** is the estimation values based on N_B_ model of grain number per rice ear.

**Figure 6** is the rice yield estimation map using the new modified water-cloud model based on panicle layer constructed in this paper. *M_wetB_* and *N*_B_, which have the smallest error with the field investigation, are selected to draw the rice yield estimation map. According to **Figure 6**, the estimated rice value at the field scale can be obtained.

### 4.2 Experimental verification

#### 4.2.1 Verification of yield estimation model

**Figure 7:**
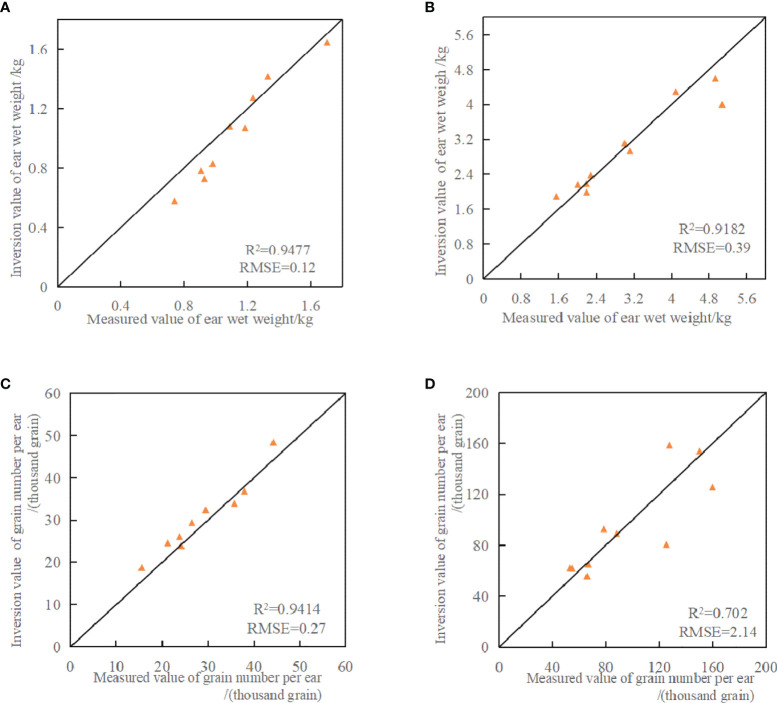
Verification diagram of inversion model of wet weight of ear and grain number per ear. **(A)** Model verification of measured values and inversion values of wet weight of rice ear M_wetB_; **(B)** Model verification of measured values and inversion values of wet weight of rice ear M_wetCDE_; **(C)** Model verification of measured values and inversion values of grain number per ear N_B_; **(D)** Model verification of measured values and inversion values of grain number per rice ear N_CDE_.

In order to verify the accuracy of the rice yield estimation model constructed in this paper, some other samples were selected for verification. For parcel B, the other 10 samples from field investigation were selected; for parcel C+D+E, the other 10 samples from field investigation were selected. **Figure 7** shows the validation of the inversion models of wet weight of ear and grain number per ear. Correlation (R^2^) and Root Mean Square Error (RMSE) were used to evaluate the accuracy of the yield estimation model.

According to **Figures 7A, B**, the inversion results of wet weight of ear were close to the measured results, and the R^2^ was 0.9477 in parcel B, with the lowest RMSE of 0.12. The R^2^ and RMSE were 0.9182 and 0.39 respectively in parcel C+D+E. This indicats that the yield estimation model constructed in this paper can accurately estimate wet weight of ear of rice. As can be seen from **Figures 7C, D**, for the inversion results of grain number per ear, the inversion results in parcel B (**Figure 7C**) were closest to the measured results, with R^2^ of 0.9414 and RMSE of 0.27. The inversion accuracy of parcel C+D+E (**Figure 7D**) was much lower than that of **Figure 7C**, with R^2^ of 0.702 and RMSE of 2.14. This error may be caused by the difference of rice varieties planted in C+D+E parcel, which resulted in different grain number per ear. And the distribution of grain number per ear per unit area is not uniform.

#### 4.2.2 Verification between estimated yield and measured yield

The rice yield estimation model constructed in this paper was used to estimate the rice yield of parcel B and parcel C+D+E, and was compared with the rice yield data of field investigation to verify the accuracy obtained in this paper. The measured data of rice yield are shown in **Table 2**. Comparison of model yield estimates with measured data is shown in **Table 3**.

**Table 2:** Field investigation data of rice yield in parcel B and C+D+E.

	Effective ear (ten thousand/hm^2^)	Total number of particles (grain/ear)	Real income (grain/ear)	The weight of 1000 grains (g)	Total output(kg/hm^2^)	yield per mu (kg/mu) (drying)
parcel B	406.5	158.9	141.2	23.6	8359.8	557.32
parcel C	363.8	451.1	279.9	25.4	10488	679.2
parcel D	357.8	439.1	349.8	26.4	11014.5	714.3
parcel E	327.7	446.6	292.3	25.3	9640.5	642.7
parcel C+D+E	349.76	445.6	307.33	25.7	10381	678.73*

*,the average yield of three fields.

**Table 3:** Verification of model yield estimate accuracy.

	Model	Estimated Ywet weight (kg/ mu) (wet)	Estimated production (kg/mu) (drying)	Real production (kg/mu)	Absolute error (kg/mu)	Relative error (%)	Precision(%)
parcel B	MwetB	854.97	589.93	557.32	32.61	5.85	94.15
	NB	——	585.0	557.32	27.68	4.97	95.03
parcel C+D+E	MwetCDE	1011.34	728.17	678.73	49.44	7.28	92.72
	NCDE	——	733.95	678.73	55.22	8.14	91.86

As can be seen from **Table 3**, for rice in parcel B, the estimated values based on wet weight of rice ear scattering model (M_wetB_) and grain number per rice ear scattering model (N_B_) were higher than the measured data. The absolute error (AE) of N_B_ was 27.68 kg/mu, the relative error (RE) was 4.97%, and the precision (P) was the highest, reaching 95.03%. The difference between model inversion yield and measured data was small. The AE, RE and P of M_wetB_ were 32.61 kg/mu, 5.85% and 94.15% respectively. For rice in parcel C+D+E, the estimated values of rice based on wet weight of rice ear scattering model (M_wetCDE_) and grain number per rice ear scattering model (N_CDE_) were still higher than the measured data, and the estimated yield of M_wetCDE_ was better than that of N_CDE_. The AE, RE and P of M_wetCDE_ were 49.44 kg/mu, 7.28% and 92.72% respectively. The AE, RE and P of N_CDE_ were 55.22 kg/mu, 8.14% and 91.86% respectively.

The reasons for the higher estimated rice yield in parcel B by the model constructed in this paper are as follows: In the functional area of grain production, only rice in parcel B was sown earlier, had good growth conditions and was fully mature. However, many grains were eaten by birds, leading to a decrease in the measured yield. Based on the analysis of the parcel C+D+E, the estimated values of the model in this paper is still higher than the measured data, because rice is in the grouting stage, which is not fully mature, and has more water content. In the process of model construction, the panicle weight was calculated according to the state of full maturity of the samples collected, while the measured yield contained some depressed grains with insufficient grout. Therefore, some reduction in production is normal.

## 5 Discussion and analysis

### 5.1 Analysis of relationship between W_1_·h_1_ and W_2_·h_2_

**Figure 8:**
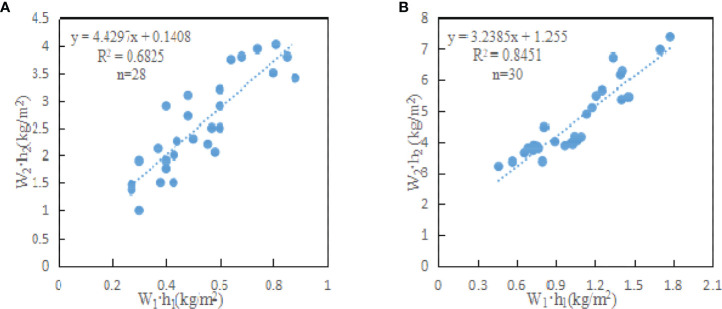
Fitting diagram of W_1_·h_1_ and W_2_·h_2_. **(A)** is the fitting diagram of W_1_·h_1_ and W_2_·h_2_ in parcel B; **(B)** is the fitting diagram of W_1_·h_1_ and W_2_·h_2_ in parcel C+D+E.

To discuss the feasibility of using the radar data with high frequency Ku band for rice yield estimation, it is necessary to explore the penetration of the radar data with Ku band in rice plants, and to further explore the relationship between the water content per unit area of panicle layer (W_1_·h_1_) and the water content per unit area of stem and leaf layer (W_2_·h_2_). According to the measured data, the fitting relationship between water content per unit area of panicle layer (W_1_·h_1_) and water content per unit area of stem and leaf layer (W_2_·h_2_) was established. The fitting relationships of parcel B and parcel C+D+E were formula (27) and formula (28), respectively. **Figure 8** shows the scatter diagram of water content per unit area of panicle layer and stem and leaf layer in different fields.

The fitting relationship of field B is:


(27) $${\left(W_{2}\cdot h_{2}\right)}_{B}=4.4297{\left(W_{1}\cdot h_{1}\right)}_{B}+0.1408\begin{array}{c} R_{B^{2}}=0.6825 \end{array}$$

The fitting relationship of field C+D+E is:


(28) $${\left(W_{2}\cdot h_{2}\right)}_{CDE}=3.2385{\left(W_{1}\cdot h_{1}\right)}_{CDE}+1.255\begin{array}{c} R_{{CDE}^{2}}=0.8451 \end{array}$$

Where, (W_1_·h_1_)_B_ and (W_1_·h_1_)_CDE_ are the water content per unit area of panicle layer (kg/m^2^) of parcel B and parcel C+D+E; (W_2_·h_2_)_B_ and (W_2_·h_2_)_CDE_ are the water content per unit area (kg/m^2^) of stem and leaf layer of parcel B and parcel C+D+E; R^2^_B_ and R^2^_CDE_ are fitting coefficients of regression formula.

According to **Figure 8**, the water content per unit area of panicle layer and stem and leaf layer of rice showed a certain linear correlation. **Figures 8A, B** both had the same trend. W_1_·h_1_ increased with the increase of W_2_·h_2_, that is, the water content of panicle layer increased with the increase of stem and leaf layer. The change of water content per unit area of parcel C+D+E (**Figure 8B**) was more obvious than that of parcel B (**Figure 8A**). Because rice in parcel B was in the fully mature stage and was about to be harvested, many leaves and stems withered, and the water content per unit area decreased significantly, which weakened the correlation between panicle layer and stem and leaf layer. The water content per unit area of panicle layer ranged from 0.3 to 0.8 (kg/m^2^), the water content per unit area of stem and leaf layer ranged from 1.5 to 4.0 (kg/m^2^), and the correlation coefficient R_B_^2^ was about 0.68. The correlation between W_1_·h_1_ and W_2_·h_2_ was good. The water content per unit area of panicle layer ranged from 0.5 to 1.7 (kg/m^2^), and the water content per unit area of stem and leaf layer ranged from 3.5 to 7.0 (kg/m^2^), with a correlation coefficient of 0.84. Therefore, it is difficult to penetrate stem and leaf layer and the pad surface layer for the SAR data with high frequency, such as Ku band. The radar echo of rice canopy is more from rice panicle layer, which effectively overcomes the shortcoming of a large amount of information of stem and leaf layer and the pad surface layer contained in low frequency data source, and reduces the difficulty of yield estimation modeling.

### 5.2 Analysis of the relationship between σ^0^ and W_1_·h_1_

**Figure 9:**
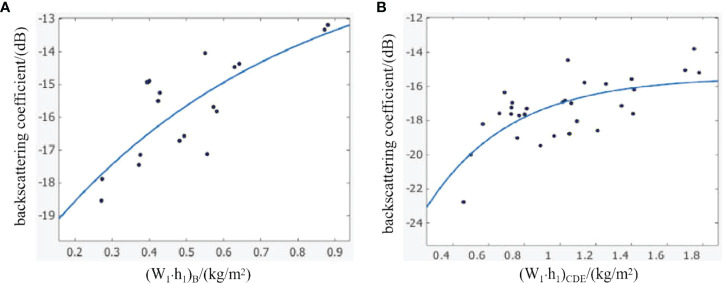
The relationship between the backscattering coefficients and W_1_·h_1_ under polarization HH. **(A)** is the relationship between the backscattering coefficients and W_1_·h_1_ under polarization HH in parcel B; **(B)** is the relationship between the backscattering coefficients and W_1_·h_1_ under polarization HH in parcel C+D+E.

According to formulas (21), (22), (27) and (28), the new modified water-cloud model at panicle layer was used to establish the relationship between SAR backscattering coefficient (σ^0^) and the water content per unit area of panicle layer (W_1_·h_1_) for parcel B and parcel C+D+E. **Figure 9** is the fitting result diagram of the model.

**Figure 9** shows that there is a certain correlation between SAR backscattering coefficient (σ^0^) and the water content per unit area of panicle layer (W_1_·h_1_), and the fitting formula is as follows:

For parcel B:


(29) $${\sigma^{0}}_{B}=-10.57-\exp(-({\left(W_{1}\cdot h_{1}\right)}_{B}+1.58)/0.6638)\begin{array}{c} {R^{2}}_{B}=0.6166 \end{array}$$

For parcel C+D+E:


(30) $${\sigma^{0}}_{CDE}=-15.44-\exp(-({\left(W_{1}\cdot h_{1}\right)}_{CDE}+1.203)/0.4669)\begin{array}{c} {R^{2}}_{CDE}=0.4708 \end{array}$$

As can be seen from **Figure 9**, the inversion results of the two models are similar, indicating that it has certain sensitivity to the change of water content in rice canopy for HH polarization mode. σ^0^ increases with the increase of W_1_·h_1_ and gradually tends to saturation. Some sample points deviate far from the fitting curve, which is mainly because the backscattering coefficient of ground objects is a range rather than a fixed value. For rice, the backscattering coefficient in the fitting result is within the allowable range. Parameter C in formula (29) and formula (30) has a great difference. Considering that rice in parcel B is already in the mature stage, and the plant height and water content per unit area of stem and leaf layer are different, so the water content per unit area of panicle layer has a great difference. And this leads that the scattering of radar wave is also different. Compared with parcel B, parcel C+D+E has higher canopy density, large leaf area and large coverage. So the parcel C+D+E has a stronger degree of attenuation of radar wave, which makes that the scattering of radar signal shows a great difference. Therefore, the fitting accuracy is lower than that of parcel B.

## 6 Conclusions

Aiming at the problem of rice yield estimation at the field scale, this paper constructed a modified water-cloud model based on panicle layer and the radar data with Ku band to estimate the rice yield. Using the UAV-based MiniSAR radar data with Ku band and the new model, the relation model of rice panicle wet weight, grain number and water content per unit area (W_1_·h_1_) was established. The yield estimation of rice in panicle and mature stage at field scale can be realized.

Through the research, some valuable conclusions can be obtained as follows:

(1) For parcel B, compared with measured data, the estimation accuracies of M_wetB_ model and N_B_ model were 95.03% and 94.15%, respectively. For parcel C+D+E, the estimation accuracies of M_wetCDE_ and N_CDE_ were more than 91.8%. The variation trend of the estimated values were basically consistent with the measured values, which indicated that the model constructed in this paper could be applied to rice yield estimation well.

(2) The accuracy of rice yield estimation is influenced by the different growth stages of rice. The method in this paper is especially suitable for rice yield estimation at the mature stage. For rice at mature stage, the estimated yield accuracy of wet weight of rice ear scattering model was almost the same as that of grain number per rice ear scattering model, both of which were over 94%. For rice at grouting stage, the yield estimation accuracy of wet weight of rice ear scattering model was 92.7% better than that of grain number per rice ear scattering model.

(3) It is difficult to penetrate stem and leaf layer to reach the pad surface layer of rice for the SAR data with high frequency, such as Ku band, so the radar echoes of rice canopy are mostly from panicle layer, which effectively overcomes the shortcoming of the radar data with low frequency contains a large amount of information about stem and leaf layer and pad surface layer. It can reduce the difficulty of yield estimation modeling. Based on the modified water-cloud model of panicle layer constructed in this paper, under HH polarization in Ku band, the estimated yield of wet weight of rice ear scattering model and grain number per rice ear scattering model is similar to the measured results, with an estimated yield accuracy of more than 91%. This model can estimate rice yield effectively, and it provides a practical method for estimating rice yield in high frequency SAR data.

The method constructed in this paper can be applied to rice yield estimation at the field scale at the mature stage of rice, and is particularly suitable for SAR data with high frequency, such as Ku band. In the study of rice yield estimation, the proposed method achieved relatively high yield estimation accuracy. However, due to the simplified processing of the model, some structural parameters of rice (such as leaf density distribution in stem and leaf layer, blade incidence, panicle angle, etc.) were not sufficiently considered, which would influence the change of rice backscattering. In this yield estimation study, only the radar data with HH polarization and Ku band from UAV-based MiniSAR was used. In the next step, the radar data with different polarization and different frequencies will be combined to carry out more accurate rice yield estimation.

## Data availability statement

The original contributions presented in the study are included in the article/supplementary material. Further inquiries can be directed to the corresponding authors.

## Author contributions

ZW wrote the manuscript, designed its structure and revised the manuscript. SW wrote the manuscript, collected and analyzed the MiniSAR data and field investigation sampling data. ZW and SW contributed equally to this work. HW contributed to the data analysis, making charts and the paper modification. LL proposed and performed the experiments, completed the acquisition of MiniSAR radar data and provided suggestions. ZL helped in collecting and analyzing the data. YZ and KW critically revised the manuscript. All authors contributed to the article and approved the submitted version.

## Funding

This research is supported by funding from the National Natural Science Foundation of China (No. 41601375, 41876202) and Natural Science Foundation of Zhejiang Province (No. LGN20D010001).

## Acknowledgments

Thanks to the Institute of Space and Space Information Innovation, Chinese Academy of Sciences for providing the MiniSAR system for this research.We want to thank the reviewers for their valuable suggestions and comments which improved the quality of the manuscript.

## Conflict of interest

The authors declare that the research was conducted in the absence of any commercial or financial relationships that could be construed as a potential conflict of interest.

## Publisher’s note

All claims expressed in this article are solely those of the authors and do not necessarily represent those of their affiliated organizations, or those of the publisher, the editors and the reviewers. Any product that may be evaluated in this article, or claim that may be made by its manufacturer, is not guaranteed or endorsed by the publisher.
